# SMS reminders to improve the tuberculosis cure rate in developing countries (TB-SMS Cameroon): a protocol of a randomised control study

**DOI:** 10.1186/1745-6215-15-35

**Published:** 2014-01-24

**Authors:** Georges Bediang, Beat Stoll, Nadia Elia, Jean-Louis Abena, Désiré Nolna, Philippe Chastonay, Antoine Geissbuhler

**Affiliations:** 1 Faculty of Medicine and Biomedical Sciences, University of Yaoundé I, PO Box 1364 Yaoundé, Cameroon; 2 Department of Radiology and Medical Informatics, University of Geneva, Geneva, Switzerland; 3 Institute of Global Health, Faculty of Medicine, University of Geneva, Geneva, Switzerland; 4 National Tuberculosis Control Programme, Ministry of Public Health, Dschang, Cameroon

**Keywords:** SMS reminder, Text messaging, mHealth, Tuberculosis, Developing country, Low-resource country, Africa

## Abstract

**Background:**

Tuberculosis is a public health problem in Cameroon, just like in many other countries in the world. The National Tuberculosis Control Programme (PNLT) put in place by the state, aims to fight tuberculosis through the implementation of international directives (Directly Observed Treatment Short, DOTS). Despite the deployment of this strategy across the world, its implementation is difficult in the context of low-resource countries. Some expected results are not achieved. In Cameroon, the cure rate for patients with sputum positive pulmonary tuberculosis (TPM+) after 6 months is only about 65%, 20% below the target. This is mainly due to poor patient adherence to treatment. By relying on the potential of mobile Health, the objective of this study is to evaluate the effect of SMS reminders on the cure rate of TPM + patients, measured using 6-month bacilloscopy.

**Methods/design:**

This is a blinded, randomised controlled multicentre study carried out in Cameroon. The research hypothesis is that sending daily SMS messages to remind patients to take their prescribed tuberculosis medication, together with the standard DOTS strategy, will increase the cure rate from 65% (control group: DOTS, no SMS intervention) to 85% (intervention group: DOTS, with SMS intervention) in a group of new TPM + patients. In accordance with each treatment centre, the participants will be randomly allocated into the two groups using a computer program: the intervention group and the control group. A member of the research team will send daily SMS messages. Study data will be collected by health professionals involved in the care of patients. Data analysis will be done by the intention-to-treat method.

**Discussion:**

The achieving of expected outcomes by the PNLT through implementation of DOTS requires several challenges. Although it has been demonstrated that the DOTS strategy is effective in the fight against tuberculosis, its application remains difficult in developing countries. This study explores the potential of mHealth to support DOTS strategy. It will gather new evidence on the effectiveness of mHealth-based interventions and SMS reminders in the improvement of treatment adherence and the cure rate of tuberculosis patients, especially in a low-resource country such as Cameroon.

**Trial registration:**

The trial is registered on the Pan-African Clinical Trials Registry (http://www.pactr.org) under unique identification number: PACTR201307000583416.

## Background

As in many other countries in the world, tuberculosis is a major public health problem in Cameroon. In 2010, there were 24,528 cases of tuberculosis (all forms included) with a prevalence rate of 122 per 100,000 inhabitants [1]. The number of new sputum positive cases in the country was 14,464 in 2010 [1]. With an estimated HIV prevalence rate of 5.3% [2], the rate of co-infection was estimated at 33% in new cases of sputum positive pulmonary tuberculosis (TPM+) [1].

In accordance with international directives (Directly Observed Treatment Short, DOTS [3] and the Global Strategy and Plan to Stop TB [4,5]), Cameroon has established the National Tuberculosis Control Programme (PNLT) for the fight against tuberculosis, financed by the state and international funding partners such as the Global Fund. This national programme aims to elaborate on and execute the National Policy in the fight against tuberculosis [1]. However, the DOTS strategy is often not well applied because its implementation, especially in developing countries, is complex [6], has numerous barriers [7] and needs enormous resources [8]. This is why there are many adaptations (Partially Observed) depending on the context. In the treatment of tuberculosis in Cameroon, for instance, during the intensive phase of treatment (the first two months), instead of receiving daily observable drug doses, a patient receives several doses weekly or monthly. In the continuation phase (from the third to the six month) they receive these doses monthly.

Irregular adherence to tuberculosis treatment causes therapeutic failure, leads to prolonged periods of infectiousness [9], provokes relapses [10], favours the emergence of drug resistance [11,12], requires prolonged and expensive treatment schemes, which do not guarantee effectiveness [13], and finally, increases morbidity and mortality [9].

The therapeutic success rate is the proportion of cases that complete 6 months of treatment and where a sputum test carried out at 5 months finds no Koch’s bacilli (KB). The cure rate is the proportion of cases that complete 6 months of treatment and where a sputum test carried out at 6 months finds no KB. In Cameroon, the therapeutic success rate is around 77% [2], while the cure rate is around 65% [2], which is 20% below the objective fixed by the PNLT. This low cure rate may be due to several causes.

It has been shown that the cure rate for tuberculosis is related to adherence to tuberculosis treatment and inversely related to HIV prevalence in the population, and to the prevalence of multi- or ultra-treatment-resistant cases [14]. According to actors in the field, one of the main causes of the low cure rate in Cameroon is non-adherence to treatment by tuberculosis patients. Also, according to these local actors, this failure in adherence itself could be due to a lack of communication and interactions between health staff and patients during the care process.

There has been a continuous expansion of mobile technologies across the world (for example, 95% of the world’s people are expected to have a mobile telephone by 2014), including developing countries. The increased use of mobile technology, such as the Short Message Service (SMS), has significant potential for health systems, and is termed mobile health (mHealth) [15,16]. SMS-based interventions involving health staff and patients can contribute to the improvement of health process and clinical outcomes [17,18]. The interventions are cost-effective both in urban and rural settings [19]. Many studies have explored the potential of mHealth (such as SMS reminders) for health objectives in the antiretroviral treatment of HIV [20-24]. Some of these interventions have been effective in improving adherence to antiretroviral treatment [23,24] and reducing viral load [23]. Effects have been observed in the prevention of HIV transmission from mother to child [25] on the adherence of health staff to guidelines for the management of malaria [26,27] and its control [28], and for the management of drug stocks [29].

For tuberculosis, studies have shown the utility of mobile phones. For example, they can be used to transmit laboratory images and results to improve notification and to reduce delays in the management of tuberculosis [30]. They can be used in the care of distant patients, as well as in the training of health staff [31]. Quite a number of studies have explored the possibility of using SMS to improve adherence to tuberculosis treatment [16,32] but none to date have looked the effect of the cure rate.

The aim of this study is to explore the potential of SMS-based reminders to improve adherence to tuberculosis treatment and to increase the cure rate in Cameroon, where adherence remains suboptimal because of a non-exhaustive implementation of DOTS.

## Methods/design

### Primary objective

The primary objective of this study is to evaluate the effect of daily SMS reminders on the cure rate of TPM + patients.

### Primary research hypothesis

Our primary hypothesis is that sending daily SMS messages to remind patients to take their prescribed tuberculosis medication, together with DOTS, will increases the cure rate (defined as the absence of KB in the sputum in a control test carried out at 6 months) from 65% (DOTS, no SMS intervention) to 85% (DOTS, with SMS intervention) in a group of adult patients newly diagnosed with TPM + in Cameroon.

### Secondary objectives

The secondary objectives of this study are to evaluate the effect of sending SMS reminders on:

•Patient adherence to tuberculosis treatment (drug prescriptions collected and doses taken).

•The punctuality of appointments during the second, fifth and sixth months as well as whether appointments were kept.

•The number of treatment failures (KB positive patients at 5 months).

•The number of participants who develop resistance (as measured by Genexpert [33]).

•The degree of satisfaction of the participants.

### Study design

**Figure 1:**
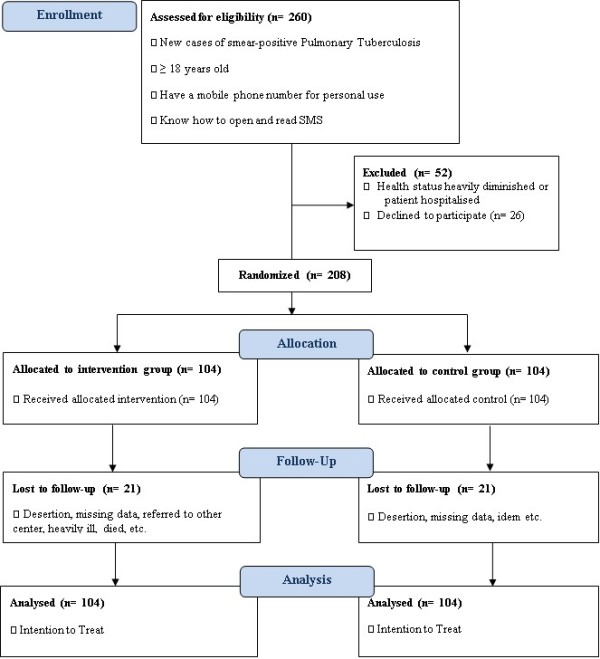
Flow diagram for the study.

This is a blinded, randomised controlled multicentre study. Patients meeting the inclusion criteria will be recruited and randomised by centre into two groups (ratio 1:1): an intervention group in which participants receive SMS reminders in addition to the usual DOTS programme versus a control group where participants follow only the usual DOTS programme (Figure 1).

The study is estimated to take about 12 months: 4 months to recruit participants and 8 months for participant follow-up. The study will be carried out in 16 Treatment and Diagnostic Centres in Yaoundé (the capital of Cameroon).

### Participants and inclusion and exclusion criteria

Participants eligible for this study must be newly diagnosed with TPM + and aged at least 18 years (born before 1 January 1995). The usual diagnostic procedure of the PNLT will be followed, which is that all patients with clinical signs of pulmonary tuberculosis will undergo two smear tests in the laboratory selected for the study. At least one of these tests must be positive. In addition, the patient must know how to read French or English, have a mobile phone for personal use, know how to open and read an SMS on a telephone and give consent (signed on the informed consent form) after having been informed about the goals, the process and the expected results of the study. Hospitalised patients or severely ill patients, as identified by health staff, will be excluded.

### Cure status

A participant is considered to be:

•Cured, if they have a negative smear test after 6 months of treatment.

•Not cured or a treatment failure if they have a positive smear test after 5 months of treatment.

•Transferred, if they are referred to continue treatment in another Treatment and Diagnostic Centre out of the study zone.

•Dead, if they die between the screening phase and the end of treatment, regardless of the cause of death.

•Lost to follow-up, if they receive at least one month of treatment and are not in any of the classes above.

### Implementation of the study

All participants in the study will receive usual care from the National Programme for the Fight against Tuberculosis. Free tuberculosis treatment lasts for an intensive phase (quadri-therapy for the first two months) followed by a continuation phase (bi-therapy from the third to the sixth month). Participants attend appointments to receive supplies of the drugs (once a week or month for the first two months then once a month between the third and sixth months) and follow-up of treatment adherence. Participants undergo a smear control test at the end of the second, fifth and sixth months. Finally, participants receive education and counselling.

After a preliminary interaction with health staff in the various centres on the importance of the study, all participants (in the two groups, control and intervention) will receive a message of welcome, then a phone call from the secretary of the study to check that the phone number actually belongs to the participant, and to confirm inclusion in the study.

#### Intervention group

The participants in the intervention group (SMS intervention) will receive daily SMS reminders in French or English. These messages will aim at reminding, inciting or motivating them to take their prescribed tuberculosis drugs. To maintain their attention and interest, these messages will be changed every two weeks (for example, ‘Good morning, it is important to take your drugs against TB every day’ , ‘Good morning, taking drugs daily increases your chance of healing’ , and so on). Additional encouraging messages will also be sent every 2 weeks (for example, ‘Congratulations! The first month of treatment is over’ , and so on). Messages are neutral and similar for both sexes. During each clinical follow-up (a visit at health centre), problems (such as side effects of drugs, problems with adherence to treatment and SMS reception) as well as solutions will be recorded by health staff in the participant’s health records and by the secretary in the daily log. Participants will also receive messages at the end of treatment to thank them for participating in the study.

#### Control group

Participants in the control group will only receive a welcome message at the beginning, to validate their entry into the study, and a message at the end of treatment to thank them for their participation. These participants will not receive daily SMS reminders.

### Outcomes and plan for analysis

#### Primary outcome

The primary objective (Table 1) is to measure the difference in cure rate at 6 months between the SMS intervention group and control group.

**Table 1:** Outcomes of the study

**Outcomes**	**Measure of outcomes**	**Type**	**Statistical analysis**
**Primary**			
**Cure**	**Percentage of cured patients**		
Bacilloscopy (second, fifth, sixth months)	Percentage of patients with positive or negative bacilloscopy results	Categorical	Chi-square
Cure (sixth month)	Percentage of cured patients	Binary	Chi-square
			Logistic regression
**Secondary**			
**Adherence of patients to treatment (second, fifth, sixth months)**			
Drug prescriptions collected and doses taken (visual analogue scale)	Percentage of adherence to treatment during the last 30 days	Continuous	*t*-test
Keeping appointments		Binary	Chi-square
Punctuality of appointments		Categorical	Chi-square
**Multi-drug-resistant tuberculosis (fifth and sixth months)**	Percentage of patients with multi-drug-resistant tuberculosis	Binary	Chi-square
**Satisfaction (sixth month)**	Satisfaction	Continuous	*t*-test

The status of each patient will be based on the results of smear tests carried out in the second, fifth and sixth months in the treatment centre. At the fifth month, two scenarios are possible. If the smear test is negative, treatment continues during the sixth month. At 6 months, the final smear test is done to see if the patient is cured. If this test is negative, the patient is classified as cured.

If the test at 5 months is positive, a second smear test will be performed to confirm the result (which is not mandatory for this study). At this moment, the patient will be considered as a treatment failure and classified as not cured in the study.

Participants lost to follow-up or referred to other treatment centres, will be considered cured (best case) or not cured (worse case) as two extreme statistical scenarios.

#### Secondary outcomes

Firstly, we will measure the impact of SMS reminders on treatment adherence (regularity in collecting prescriptions and the percentage of prescribed doses taken; Table 1). Treatment adherence will be estimated by patient self-evaluation (indirect method) based on the visual analogue scale (VAS) during follow-up visits at 2, 5 and 6 months. Used in several studies [20,34-37], VAS measures the adherence of patients to treatment and correlates with results obtained from more objective methods such as electronic follow-ups [38]. Treatment adherence will be assessed by asking the patients to estimate their adherence to treatment on a 0 to 100% scale during the last 30 days before an appointment. This will give an adherence score and will be recorded three times (at the second, fifth and sixth months) by health staff responsible for the follow-up of these patients. We intend to use the average of the three values as a continuous variable to reflect patient global adherence to treatment. Further, our analysis will try to determine whether good adherence improves the cure rate.

Secondly, we will evaluate how well participants keep appointments at the second, fifth and sixth months. We will use a binary variable: ‘yes’ for patients who come between 4 days before and 4 days after the appointment date and ‘no’ for those who do not. We will use a categorical variable: ‘early’ for participants who come more than 4 days before the appointment date, ‘prompt’ for participants who come between 4 days before and 4 days after the appointment date and ‘late’ for participants who come more than 4 days after the appointment date.

Thirdly, we will estimate and compare the number of treatment failures due to resistance to tuberculosis drugs between the two groups. The laboratory test (resistance to rifampicin as measured using Genexpert [33]) will be conducted after the fifth month for defaulters by the PNLT. This variable will be analysed as a binary variable.

Finally, we will compare the degree of satisfaction for the two groups at the third appointment (at the end of 6 months). This assessment of the degree of satisfaction will be made by health staff involved in the care of participants using a questionnaire.

### Sample size

Presently, the cure rate for tuberculosis with the DOTS strategy is around 65% [2] in Cameroon, about 20% lower than the objective fixed by PNLT. Our hypothesis is that the cure rate in the SMS intervention group will reach 85%. For an attribution ratio of 1:1 in the two groups, 80% power, an alpha error of 5% and a two-sided test (effects may be interpreted independent of the way they go), we require a sample size of at least 166 participants [39], 83 in the SMS intervention group and 83 in the control group. Considering that the risk of loss to follow-up at the end of the study is important, we increased the sample size by 20%, giving 199 participants. Also, for the urban zone, we estimated that at least 90% of eligible people will have mobile phones and that at least 80% of them will consent to take part. The total number of eligible participants was therefore increased by 10% and another 20%, leading to a total of 260 participants.

### Randomisation

In the first step, participants will be stratified according to recruitment centre. The different treatment centres will be alphabetically coded (A, B, C and so on) and participants attached to each centre will be given numerical codes (for example, patients in centre A will be numbered A001, A002 and so on).

Randomisation will be carried out using a computer program by the main investigator. Participants who are eligible and who have consented to participate in the study will be registered. Their study identifier and telephone number will be communicated to the main investigator and to the secretary, for their randomly allocation by centre into one of the two groups (intervention or control) and for the continuous updating of the mailing of SMS respectively.

The health staff in the hospitals will not be involved in the allocation of participants into groups. This prevents allocation bias and minimises the risk of selection bias (simple blind).

### Feasibility of the study

The number of new cases of TPM + in Cameroon was 14,464 in 2010, that is an average of 3,616 new cases per trimester. The Littoral (22%) and Centre (20%) regions have half of the new cases, mainly in Douala 17% (the economic capital) and Yaoundé 13% (the political capital). By recruiting participants only in Yaoundé, we will reach 13% of the 3,616 new cases of TPM + in a trimester, that is 471. A recruitment phase of 4 months should therefore be long enough to recruit a sufficient number of participants to the study.

### Data collection

Health staff taking care of participants in the study will collect data using patient follow-up files developed by the research team. These will contain all the necessary questionnaires, scales and lists of problems. The follow-up files will be kept with the participants’ health records. A marker will be placed on the file of each participant to reduce the risk of them being ‘forgotten’ by health staff during the study. Forms in the file will be completed in French for each visit (the day of inclusion and the second, fifth and sixth months).

Before the onset of recruitment, a meeting will be held with the staff members of the treatment centres. This will inform them of and validate the strategies for the implementation of the study (inclusion of participants, collection of data, use of VAS by patients to evaluate adherence, transmission of data and so on).

During the recruitment phase, a secretary will be responsible for collecting telephone numbers from the participant study files twice a week from each Treatment and Diagnostic Centre. These will be entered into the SMS database. The secretary will also collect socio-demographic data (from the day of inclusion), smear test results (from the day of inclusion, and the second, fifth and sixth months), punctuality data and results of resistance to rifampicin tests (Genexpert).

At least two sessions to supervise staff members (2 months and 6 months after the onset of recruitment) will be carried out in each centre so that data collection remains effective and consistent. Treatment centres facing problems will receive additional training and supervision at appropriate intervals. After recruitment, the secretary will collect data once a week to keep statistics and participant registers up to date. At the final follow-up (during the third visit at the end of the sixth month), health staff will administer a self-evaluation questionnaire on the degree of satisfaction to the participants.

### Plan for data analysis

The results of this study will be reported following CONSORT standards [40] on randomized trials. A data entry mask developed using the Epi Data Entry program by the main investigator will be used to enter and control the quality of patient data. Analysis of the primary outcome will use the intention-to-treat approach, which includes all participants in their initial study group allocation. All participants for which there are no smear test results at 6 months will be considered not cured or cured (worst case or best case scenario). Results will be presented depending on the type of variable: means (± standard deviation) and medians (minimum and maximum) for continuous variables and percentages for categorical or binary variables.

The two groups will be described according to their baseline characteristics. Student’s test (*t*-test) will be used to calculate the difference between the two groups for continuous variables while the chi-square test will be used for categorical or binary variables. The association between study groups and cure rate will be analysed using a logistic regression model reporting the odds ratio and 95% CI.

Each individual characteristic, such as age, sex, level of education, socio-economic category, and HIV status, will be tested for an association with the cure status in a univariate analysis of the control group. Each characteristic that is significantly related with the outcome (*P* < 0.1) will be tested using a bivariate model that includes the study group, cure status and the potential confounder. If introducing the variable changes the odds ratio by more than 10%, then the variable will be considered as a potential confounder of the association and will be used in the final multivariate model.

The data from this study will be analysed using the SPSS programme (v21.0) or STATA (v11). Comparisons between the two groups will be expressed in terms of the odds ratio and the number needed to treat. Open questions in the questionnaire (especially problems observed during follow-up) will be analysed using the content analysis technique.

### Ethical considerations

This study strictly respects the ethical principles of the Helsinki declaration [41] and local recommendations for medical research. The protocol was submitted to the National Ethical Committee of Cameroon and has been approved. It also received administrative authorisation from the Ministry of Public Health of Cameroon. All eligible participants will be contacted by health staff involved in the study. Before signing the consent form, they will receive key information on the study: objectives, data collection, expected results, duration, benefits, voluntary character, the right to withdraw at any time, confidentiality, use of data and results obtained. Data from participants collected by health staff and the secretary will be treated as confidential and retained in their health records. Patient telephone numbers will be used only for sending SMS messages and will not be communicated to any person not involved in the study. SMS messages will be strictly confidential. The results of the study will be published respecting the strict anonymity of all participants. Patients will be informed on the main results of the study through SMS messages. Continuous training and information sessions will be organised for collaborators regarding the results of the study. At the end of the study, the list of SMS message sent and patient health records will simply be destroyed. They will not be used for any other purpose.

### Material and equipment for sending SMS messages

A proprietary application will be used to send regular SMS messages to participants through the web [42]. The program logs SMS messages and produce reports of those sent.

## Discussion

The PNLT has fixed specific objectives for the fight against tuberculosis in Cameroon: to screen at least 70% of TPM + cases, to cure at least 85% of people diagnosed TPM + using standardised treatment, to protect at least 80% of the children born each year through vaccination (Bacillus Calmette-Guérin or BCG), to ensure the availability of chemotherapy for children younger than five years who have had contact with contagious people and to ensure the availability of chemoprophylaxis for people living with HIV [1]. These objectives raise numerous challenges in the fight against tuberculosis, in terms of infrastructure, organisation, socio-political context and funding [43,44]. The challenges for public health are the capacity of the programme to detect a large number of TPM + cases, to maintain people in the programme and to cure most of them to prevent drug resistance [43]. To attain these objectives, Cameroon has implemented the World Health Organisation’s DOTS [6] strategy. This ensures the best monitoring and prolonged contact of patients with the health system.

Although it has been demonstrated that the DOTS strategy produces a significant improvement in the fight against tuberculosis [45], its application remains difficult in developing countries. Through its main objective, this study has the potential to support the DOTS strategy in achieving the expected outcomes. This is a relatively new kind of intervention, which is increasingly being used in health systems. Conclusive results have been observed in other areas, like HIV/AIDS, where it has been demonstrated that SMS reminders are effective in improving adherence to antiretroviral treatment [23,24] and reducing viral load [23].

This study seeks to provide new evidence on the effectiveness of interventions based on mHealth and on the use of SMS reminders to improve treatment adherence and the cure rate of tuberculosis patients. If positive, the results from this study may be used to reinforce programmes in the fight against tuberculosis in Cameroon as well as in other settings. If negative, the results will help to identify why the intervention did not work.

Nevertheless, the realisation of this type of study in a resource-limited setting needs to overcome many challenges and difficulties. These aspects will be addressed in the next paper on this study.

## Trial status

The recruitment of patients began on 25 February 2013. We distributed 400 questionnaires in 16 tuberculosis treatment centres. Currently only 12 centres have begun recruiting patients. Of the 260 eligible patients required, 288 patients have now been included. To date, the recruitment of participants was stopped.

## Abbreviations

DOTS: Directly observed treatment short; HIV: Human immunodeficiency virus; KB: Koch’s bacillus; mHealth: mobile health; PNLT: Programme National de Lutte contre la Tuberculose au Cameroun (National Tuberculosis Control Programme); SMS: Short Message Service; TB: Tuberculosis; TPM+: Sputum positive pulmonary tuberculosis; VAS: visual analogue scale.

## Competing interests

The authors declare that they have no competing interests.

## Authors' contributions

GB was involved in the conception of the study, its implementation and the writing and editing of this protocol. BS was involved in the conception of the study and the writing and revising of this research article. NE was involved in the conception of the study and the revising of this research article. JLA and DN were involved in the implementation of the study and revising this protocol. PC was involved in the conception of the study and revising this protocol. AG was involved in the conception of the study, support and revising this protocol. GB and BS translated the final French version of the paper into English. GB, BS, NE and AG corrected the English version and approved it. All authors read and approved the final manuscript.

## Authors’ information

GB is a medical doctor, with a Master of Advanced Studies in Medical Informatics and a Master of Advanced Studies in Public Health from the Faculty of Medicine, University of Geneva. BS is a medical doctor with an MPH from the Institute of Social and Preventive Medicine, Faculty of Medicine, University of Geneva. NE is a medical doctor with an MSc in Epidemiology from the Institute of Social and Preventive Medicine, Faculty of Medicine, University of Geneva. JLA is a medical doctor with an MPH and is a member of the Cameroon National Programme for the Fight against Tuberculosis. DN is a medical doctor with an MPH and is a member of the Cameroon National Programme for the Fight against Tuberculosis. PC is a medical doctor, a professor and the head of the Institute of Social and Preventive Medicine, Faculty of Medicine, University of Geneva. AG is a medical doctor, a professor and the head of the Department of Medical Informatics and Radiology, University of Geneva.
